# Changes in Premenstrual Syndrome Severity, Nutrition Knowledge, and Eating Attitudes Following Online Nutrition Education in Female University Students: An Uncontrolled Single-Arm Pretest–Posttest Study

**DOI:** 10.3390/nu18172893

**Published:** 2026-09-03

**Authors:** Esra Tansu Sarıyer, Murat Baş, Hatice Çolak Çetinkaya

**Affiliations:** 1 Department of Nutrition and Dietetics, Graduate School of Health Sciences, Acibadem Mehmet Ali Aydinlar University, 34752 Istanbul, Turkey; dytesrasariyer@gmail.com; 2 Department of Nutrition and Dietetics, Hamidiye Faculty of Health Science, University of Health Sciences, 34668 Istanbul, Turkey; 3 Department of Nutrition and Dietetics, Faculty of Health Sciences, Acibadem Mehmet Ali Aydinlar University, 34752 Istanbul, Turkey; 4 Department of Nutrition and Dietetics, Institute of Health Sciences, Erciyes University, 38030 Kayseri, Turkey; colakhatice5@gmail.com

**Keywords:** premenstrual syndrome, nutrition education, eating attitudes, nutrition knowledge, university students, single-arm study

## Abstract

**Background/Objectives:** This study evaluated changes in PMS severity, nutrition knowledge, and eating attitudes over a 6-week online nutrition education programme delivered to female university students. **Methods:** This uncontrolled single-arm pretest/posttest study included 112 female university students recruited by convenience sampling from a women’s dormitory. Nutrition education was delivered daily for 6 weeks via WhatsApp as 42 informational cards. Measurements were taken before and after the programme. Attitudes and behaviours associated with disordered eating were screened using the Eating Attitudes Test-26 (EAT-26), nutrition knowledge using the Nutrition Knowledge Level Scale for Adults (NKLSA), and premenstrual symptom severity using the Premenstrual Syndrome Scale (PMSS). **Results:** No significant changes occurred in nutrition knowledge (*p* = 0.409) or eating attitudes (*p* = 0.654). The PMSS score significantly decreased (mean change = 14.28 ± 46.34; 95% CI 5.60–22.96; *p* = 0.001, d_z_ = 0.31, 95% CI 0.12–0.50), with decreases across eight subscales (d_z_ = 0.19–0.38; BH q < 0.05), whereas appetite changes were unchanged (*p* = 0.423); the largest standardized changes were observed for depressive mood and bloating. PMSS change was not correlated with knowledge (r = 0.001) or knowledge with eating attitudes (r = 0.057); PMSS and eating attitude changes were weakly positively correlated (r = 0.187, *p* = 0.048; exploratory, not corrected for multiplicity). **Conclusions:** Following a 6-week WhatsApp-based nutrition education programme, self-reported PMS severity decreased, particularly mood and somatic symptoms, without changes in nutrition knowledge or eating attitudes. The uncontrolled single-arm design precludes attributing these changes to the programme.

## 1. Introduction

Premenstrual syndrome (PMS) is defined as a cyclical pattern of physical or mood-related symptoms, or a combination of both, that occurs during the luteal phase and resolves during or immediately following menstruation [1,2]. The global prevalence of PMS among females of reproductive age is 47.8% [1]. Approximately 20% of these females experience symptoms severe enough to interfere with their daily activities, whereas the remainder experience mild-to-moderate symptoms [1,2]. PMS symptoms encompass appetite changes, weight gain, abdominal pain, back pain, lower back pain, headaches, breast swelling and tenderness, nausea, constipation, anxiety, irritability, anger, fatigue, restlessness, mood swings, and crying [1,2]. Although its etiology is not well elucidated, a complex interaction of biological, metabolic, and psychosocial factors plays a role in its development [3]. The most widely accepted view is that it is caused by an imbalance between neurotransmitters, including serotonin and dopamine, in the central nervous system and gonadal steroids, including estrogen and progesterone [3]. In this regard, fluid retention due to the impact on the renin–angiotensin–aldosterone system, micronutrient deficiencies, stress, and genetic and psychosocial factors also play a role [3]. Some studies have demonstrated an association between diet quality, dietary patterns, and the presence and severity of PMS [4,5,6,7]. Moreover, PMS has been specifically associated with increases (e.g., sugar, saturated fat) or decreases (e.g., dietary fiber, vitamin E, complex carbohydrates) in the intake of certain nutrients [4,5,6,7].

Eating attitudes refer to the totality of nutritional behaviors that develop from birth, become established over time, and take on an individual-specific character. Food attitudes, encompassing food-related beliefs, thoughts, emotions, and behaviors, are considered a complex phenomenon influenced by motor, cognitive, social, and emotional developmental processes, as well as environmental factors [8,9]. The literature suggests that improvements in eating attitudes and dietary quality (a diet rich in fruits, vegetables, whole grains, and calcium) can be one of the most significant nonpharmacological approaches to managing PMS [10,11,12]. By contrast, skipping meals and irregular eating habits can exacerbate PMS symptoms [10,11,12].

Nutrition knowledge comprises information on nutrition, food groups, and the content of nutrients. Higher nutrition knowledge has been associated with more favourable food choices, although knowledge alone does not necessarily translate into behaviour change [13,14,15]. Limited nutrition knowledge may contribute to less favourable food choices, which can directly impact health, demonstrating the influence of nutrition on health. Particularly during college years, when dietary habits are formed, the level of nutrition knowledge represents a factor with a significant impact, ranging from societal dietary habits to public health [13,14,15]. The effectiveness of nutrition education is crucial for enhancing nutrition knowledge. Although face-to-face instruction is one of the most effective methods in nutrition education, online education is also proving effective and becoming increasingly popular, particularly among young adults, owing to heightened internet and social media use [14,16].

This study aimed to evaluate changes in the severity of PMS, the level of nutrition knowledge, and eating attitudes among female students living in a dormitory over a 6-week period during which online nutrition education was delivered. In this context, the study also sought to examine the association between changes in premenstrual symptom severity and changes in nutrition knowledge and eating attitudes. Given the absence of a control group, this study focused on pre–post changes rather than intervention efficacy.

## 2. Materials and Methods

### 2.1. Study Design and Setting

**Figure 1:**
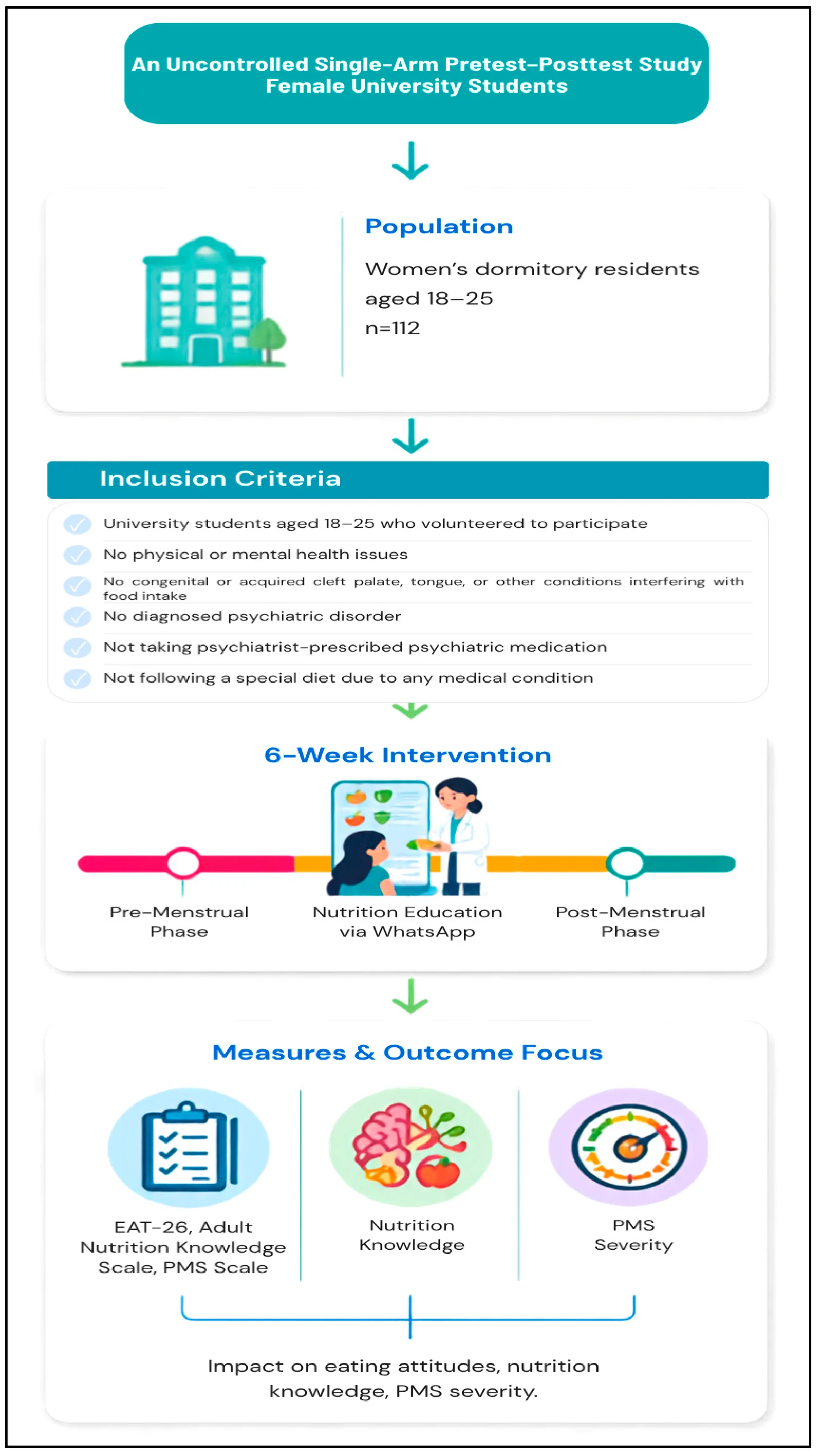
Sample selection and study process.

This study was designed as a single-arm pretest/posttest (quasi-experimental) intervention study without a control or comparison group and was conducted by collecting face-to-face data from female college students residing in a women’s dormitory in Istanbul, Türkiye, between March 2026 and June 2026. Reporting follows the TREND statement for non-randomized evaluations of behavioural interventions. The methodology is summarised in Figure 1.

### 2.2. Participants, Sampling and Eligibility Criteria

The subjects were female dormitory residents who voluntarily agreed to participate following the posting of written notices in the dormitory and an invitation through the dormitory messaging group. Overall, 230 females aged 18–25 were eligible, 185 accepted the invitation to participate, 32 were excluded due to failure to meet the eligibility criteria, 41 declined to participate or did not participate in the initial assessment and 112 completed the initial and the follow-up assessments. All 112 who submitted preliminary data did not complete the follow-up evaluation.

The following were the inclusion criteria: being a university student aged 18–25 years who volunteered to participate in this study; having no physical or mental health issues; and not following a special diet due to any medical condition. Participants were excluded if they had a diagnosed congenital or acquired cleft palate, tongue abnormality, or any other condition that would interfere with eating, or if they had a diagnosed mental health disorder or were taking psychiatric medication prescribed by a psychiatrist. Exclusion criteria were conditions that affected oral intake and oral processing, as these might independently limit a participant’s ability to change food choices after nutritional education and might change dietary intake, confounding the desired results. Psychiatric diagnoses and use of psychotropic medications were also exclusion criteria because both are known to affect premenstrual symptom reporting, appetite, and eating behaviors.

### 2.3. Sample Size

The sample size was calculated using the sample size formula for a known population (*n* = Nt^2^pq/[d^2^(N − 1) + t^2^pq]), on the basis of a 0.05 margin of error and a 95% confidence interval. This formula is used to determine the number of participants needed to estimate the proportion of students who reach the threshold of the PMSS for PMS with a certain accuracy and does not constitute a power analysis for a pre–post comparison. Thus, an additional analysis for the main result was conducted: a paired-samples *t*-test with α = 0.05 (two-tailed) and 80% power is needed with 88 participants to detect a standardized within-person effect of d_z_ = 0.30. The final sample of 112 individuals provided 80% power to detect d_z_ ≥ 0.26 for the primary outcome (post hoc power for the observed d_z_ = 0.31 was 0.90), but it was underpowered for smaller subscale effects and for weak correlations (r < 0.26).

### 2.4. Ethical Approval

This study was conducted following the guidelines of the Declaration of Helsinki 2013. The Hamidiye Scientific Research Ethics Committee of the Turkish Republic University of Health Sciences approved this study (approval number: 26/43). All participants returned written informed consent before the intervention.

### 2.5. Data Collection Tools

Measurements were taken before and after the 6-week training period. The following measures were used: the Eating Attitudes Test-26 (EAT-26), the Nutrition Knowledge Level Scale for Adults (NKLSA), and the Premenstrual Syndrome Scale (PMSS). The primary outcome was the total score of the PMSS. Secondary outcomes were nutrition knowledge (NKLSA), eating attitudes (EAT-26), and percentage of participants meeting the PMSS threshold. PMSS subscales (nine) were predefined secondary outcomes assessed with multiplicity adjustments. No assessment of dietary intake or diet quality was carried out, so diet quality was not reported as an outcome.

#### 2.5.1. Sociodemographic Characteristic-Related Questions

This section comprises questions regarding the participant’s age, sex, height, body weight, current diagnosed medical conditions, medication use, menstrual cycle frequency, and age at menarche, as well as questions about snacking and meal habits. Height and body weight were measured by the researchers, and body mass index (BMI) was calculated as weight (kg)/height (m)^2^. Tobacco and alcohol use were recorded as present/absent.

#### 2.5.2. EAT-26

This is the 26-item short form of the EAT-40, originally developed by Garner and Garfinkel (1979) [17] and later revised and shortened by Garner, Olmsted, Bohr, and Garfinkel (1982) [18]. In 2019, Ergüney–Okumuş and Sertel–Berk performed the validity and reliability study of the scale in Turkish. The scale is scored as follows: 3 = Always, 2 = Very often, 1 = Often, 0 = Other responses (Sometimes, Rarely, Never). The first 25 items are normally coded, whereas the 26th item is reverse-coded [17,18,19]. The EAT-26 is a screening instrument for attitudes and behaviours associated with disordered eating; it is not a measure of general dietary behaviour, diet quality or “healthy eating”. EAT-26 scores are therefore interpreted throughout this manuscript only as an index of disordered-eating risk. In the present sample, Cronbach’s α was 0.857 at baseline and 0.873 at follow-up.

#### 2.5.3. NKLSA

This scale is employed for measuring individuals’ knowledge levels regarding basic nutrition, nutrient content, and the association between nutrition and health. This 20-item scale is employed for measuring basic nutrition and nutrition–health association levels [20]. In the present sample, Cronbach’s α was 0.594 at baseline and 0.534 at follow-up.

#### 2.5.4. PMSS

Developed by Gençdoğan in 2006 based on the Diagnostic and Statistical Manual of Mental Disorders, Third Edition and Diagnostic and Statistical Manual of Mental Disorders, Fourth Edition, Text Revision, this scale is designed to assess the severity of premenstrual symptoms. The PMSS, a five-point Likert-type scale, comprises nine subdimensions (depressive mood, anxiety, fatigue, irritability, depressive thoughts, pain, appetite changes, sleep changes, and bloating) [21]. A total score of ≥132 points is used as a threshold indicating a high premenstrual symptom burden.

The PMSS is a retrospective, single-time assessment tool, whereas for premenstrual disorders, prospective daily symptom tracking over a minimum of two consecutive cycles is recommended [22]. Because of the cyclical nature of PMS, the timing of assessment in relation to the luteal phase is a key factor affecting scores. For all subjects in the present study, the first and second assessments were fixed to calendar dates and were 6 weeks apart and not timed to the same phase of the menstrual cycle for each individual; the cycle day or phase at each assessment was not recorded. Therefore, we consistently refer to being above the PMSS threshold as “meeting the PMSS threshold for PMS” and this should not be taken as a clinical diagnosis or as a true representation of the incidence of PMS or premenstrual dysphoric disorder. In the present sample, Cronbach’s α for the PMSS total score was 0.972 at baseline and 0.978 at follow-up. Also, Cronbach’s alpha coefficients for the PMSS subscales ranged from 0.819 to 0.937 at baseline and from 0.843 to 0.964 at follow-up.

### 2.6. Nutrition Education Intervention

In this study, participants received nutrition education via WhatsApp for 6 weeks. A single WhatsApp group was created for the participating students, and one informational flashcard was shared each day, giving 42 cards in total. The education included cards containing information from scientific sources on healthy eating, nutrition during menstruation, pre- and postmenstrual nutrition, and nutritional strategies for alleviating PMS symptoms. Researchers shared one informational poster per day with this group regarding healthy and balanced nutrition during the premenstrual, menstrual, and postmenstrual periods. The main educational objectives were that, upon completion of the program, participants would be able to (i) describe the basics of a sufficient and balanced diet, (ii) identify dietary factors associated with the severity of premenstrual symptoms, (iii) recognize the relationship between meal regularity, meal skipping and premenstrual symptoms, and (iv) mention practical dietary strategies applicable to the premenstrual, menstrual and postmenstrual phases. The material was organized into six weekly themes. Week 1: The basics of adequate and equitable nutrition. Week 2: The menstrual cycle and nutritional requirements in each phase. Week 3: Micronutrients that have been linked to premenstrual symptoms (i.e., calcium, magnesium, vitamin B6, vitamin D). Week 4: Dietary habits, consistency of meals, and skipping meals. Week 5: Nutrition during the premenstrual and menstrual phases and how to address specific symptoms. Week 6: Putting it all together, practical meal planning, and frequently asked questions. The full set of the card content is included in Appendix A Table A1.

The content of the shared posters was prepared based on current and reliable scientific sources [22,23,24,25] by three authors who are dietitians and academicians. Cards containing statements that could not be verified against the source documents were revised or discarded.

**Figure A1:**
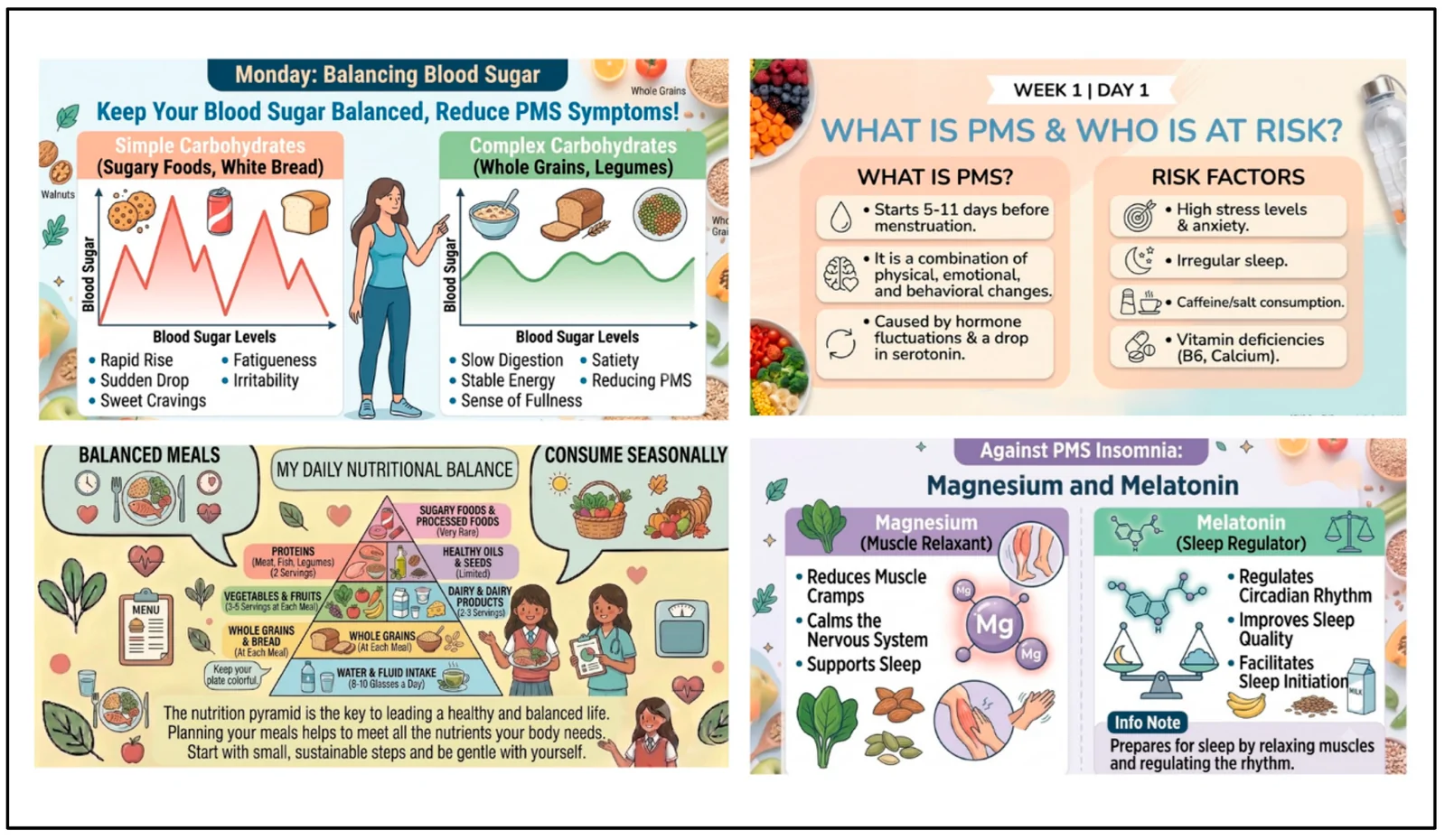
Nutrition education flashcards. Representative cards from the 42–card set delivered via WhatsApp. Card images were rendered with a generative tool from scientific content written and verified by the research team; every card was checked against the source documents and approved before delivery.

This training programme spanned 6 weeks to cover the pre- and postmenstrual phases. To assess changes over the programme, a pretest/posttest method was employed before and after the intervention. A weekly interactive question-and-answer session was offered within the WhatsApp group throughout the programme; participation was voluntary and no questions were submitted by participants. The scientific content of each card was composed by the researchers, and the images were created using the Google Gemini generative tool (Nano Banana 2 version; Gemini 3.1 Flash Image; Google LLC, Mountain View, CA, USA). Google Gemini was used only to create the visual elements of the cards based on content prepared by the research team. It was not used to generate the scientific content of the educational materials. Cross-checking of all cards produced, including all claims regarding nutrients, hormones, melatonin, magnesium and symptom relief, against source documents [22,23,24,25] was performed, along with approval by the reviewing dietitian(s) before distribution. Examples of the flashcards are provided in Appendix A Figure A1. The program’s engagement was not tracked in a systematic way. Message transmission and acknowledgment, number of cards accessed by each participant, and attendance at the optional weekly question and answer session were not monitored, leading to a lack of objective adherence metric. This limitation is addressed in Section 5.

### 2.7. Statistical Analysis

Statistical analyses were performed using IBM SPSS Statistics for Windows, Version 27.0 [26]. Descriptive statistics for categorical variables were presented as frequencies and percentages, whereas continuous variables were expressed as means ± standard deviations (SDs). The normality of continuous variables was assessed using skewness and kurtosis values (acceptable range, −2 to +2). Comparisons between pre- and posttest variables were performed using the paired samples *t*-test. Comparison of the proportion of participants meeting the PMSS threshold between pre- and posttests was performed using the McNemar exact test. Associations between changes in scale scores were assessed using Pearson correlation analyses. All statistical tests were two-tailed; a *p*-value of <0.05 was considered statistically significant [27].

An analytical assessment hierarchy was used. The single primary comparison was the change in the total PMSS score, assessed without any correction at the α = 0.05 level. Secondary confirmatory analyses were the changes in the total NKLSA and EAT-26 scores and the percentage of those who reached the PMSS threshold. The nine PMSS subscales were a second set of tests, with *p*-values adjusted using the Benjamini–Hochberg false discovery rate method (q-values are given in Table 2). The correlation analyses shown in Table 4 and the subgroup analyses shown in Table 5 are preliminary analyses uncorrected for hypothesis generation purposes only and for multiple comparisons. Effect sizes are reported as Cohen’s d_z_ for paired observations and are calculated by dividing the mean of the within-person difference scores by the standard deviation of the difference scores (d_z_ = M_diff_/SD_diff_). Correlation between pre- and post-test scores was not considered in this calculation. Mean changes and d_z_ values are reported along with two-sided 95% confidence intervals.

## 3. Results

Overall, 112 females participated in this study; most participants were single (94.6%) and received a bachelor’s degree (78.6%). Smoking was reported by 27.7% of the participants, and alcohol consumption by 16.1% (Table 1).

Of the participants, the mean age, body mass index, and daily water intake were 22.76 ± 2.81 years, 22.90 ± 3.74 kg/m^2^, and 1.51 ± 0.66 L, respectively. The mean number of main meals per day was 2.18 ± 0.47; the mean number of snacks per day was 1.38 ± 0.85. The mean age at menarche, menstrual cycle length, and menstrual bleeding duration were 13.06 ± 1.26 years, 28.97 ± 7.29 days, and 6.26 ± 1.22 days, respectively (Table 1).

The comparison of pre- and postonline nutrition education scale scores is presented in Table 2. No statistically significant difference was observed between pre- and posteducation NKLSA total scores (53.20 ± 7.16 vs. 52.65 ± 6.18; mean change 0.54, 95% CI −0.76 to 1.84; *p* = 0.409). Similarly, no significant change was noted in the EAT-26 total score (10.68 ± 9.60 vs. 11.16 ± 10.48; mean change −0.48, 95% CI −2.61 to 1.65; *p* = 0.654).

By contrast, the PMSS total score, the primary outcome, significantly declined following the education (mean change = 14.28 ± 46.34 points; 95% CI 5.60 to 22.96; *p* = 0.001, Cohen’s d_z_ = 0.31, 95% CI 0.12 to 0.50). Investigating the PMSS subscales revealed decreases that remained significant after Benjamini–Hochberg correction in depressive mood (*p* < 0.001; q = 0.001; d_z_ = 0.38), anxiety (*p* = 0.009; q = 0.017; d_z_ = 0.25), fatigue (*p* = 0.042; q = 0.047; d_z_ = 0.19), irritability (*p* = 0.021; q = 0.031; d_z_ = 0.22), depressive thoughts (*p* = 0.005; q = 0.011; d_z_ = 0.27), pain (*p* = 0.002; q = 0.006; d_z_ = 0.30), sleep changes (*p* = 0.041; q = 0.047; d_z_ = 0.20), and bloating (*p* = 0.001; q = 0.003; d_z_ = 0.33). By contrast, the appetite changes subscale demonstrated no significant change (*p* = 0.423; q = 0.428) (Table 2). Under the more conservative Holm–Bonferroni procedure, the fatigue (adjusted *p* = 0.122), irritability (adjusted *p* = 0.084) and sleep-change (adjusted *p* = 0.122) findings would not reach the conventional significance threshold.

The effect sizes for the PMSS total score and subscales, where significant differences were noted, ranged from small to small-to-moderate (Cohen’s d_z_ = 0.19–0.38), with the numerically largest values observed in the depressive mood (d_z_ = 0.38) and bloating (d_z_ = 0.33) subscales; the 95% confidence intervals of all subscale effect sizes overlapped substantially, so no subscale can be identified as differentially responsive (Table 2).

The proportion of participants meeting the PMSS threshold for PMS was 58.9% (*n* = 66) before the training and 49.1% (*n* = 55) afterward, a difference of 9.8 percentage points that did not reach statistical significance (*p* = 0.108) (Table 3).

No significant association was noted between the ΔPMSS and ΔNKLSA scores (r = 0.001, *p* = 0.994). Similarly, no significant correlation was noted between the ΔNKLSA and ΔEAT-26 scores (r = 0.057, *p* = 0.547). By contrast, a weak but statistically significant positive correlation was observed between ΔPMSS and ΔEAT-26 scores (r = 0.187, *p* = 0.048) (Table 4). This correlation explains approximately 3.5% of the variance, was one of several exploratory tests, and would not survive correction for multiple comparisons; it is therefore reported as hypothesis-generating only.

### Exploratory Subgroup Analyses

Exploratory subgroup analyses of the change in PMSS total score were performed for tobacco consumption (yes/no) and BMI classification (<18.5, 18.5–24.9, ≥25.0 kg/m^2^). These analyses were not pre-specified, not adjusted for multiplicity, and are underpowered; they are hypothesis-generating only (Table 5).

A significant reduction in total PMSS scores was observed in the exploratory subgroup analysis for those participants abstinent from tobacco (mean change: 12.35; 95% CI 3.02–21.67; *p* = 0.010; d_z_ = 0.29) but not tobacco users (*p* = 0.065). Based on BMI categories, the group with a BMI less than 18.5 kg/m^2^ had a significant reduction in the PMSS total score (mean change: 36.23, 95% CI: 7.22–65.24; *p* = *0*.019; d_z_ = 0.75). Decreases in participants with BMI of 18.5–24.9 kg/m^2^ (*p* = 0.059) and BMI of ≥25.0 kg/m^2^ (*p* = 0.123) were not statistically significant.

## 4. Discussion

This uncontrolled single-arm study evaluated changes in PMS severity, nutrition knowledge, and eating attitudes among female college students over a 6-week period during which a nutrition education program was delivered via the widely used WhatsApp application. The findings indicated that, following the 6-week nutrition education program, no statistically significant change was noted in the participants’ nutrition knowledge levels or general eating attitudes; however, the severity of self-reported PMS symptoms was significantly reduced. The analysis of subdimensions showed significant reductions in different areas (depressive mood, anxiety, fatigue, irritability, depressive thoughts, pain, sleep disturbances and bloating), except for appetite changes. The subdimensions depressive mood and bloating showed the largest standardized variations. Furthermore, an exploratory analysis revealed a positive, statistically significant but weak correlation between changes in PMS severity and changes in eating attitude scores. Because the study did not include a control or attention-control group, the observed changes cannot be directly attributed to the educational program. Natural variation in premenstrual symptoms between cycles, regression to the mean, repeated questionnaire administration, expectancy or Hawthorne effects, and other uncontrolled factors may have contributed to the findings.

The results of this study on average age at menarche (13.06 ± 1.26 years), average duration of menstrual cycle (28.97 ± 7.29 days) and average duration of bleeding (6.26 ± 1.22 days) were consistent with the ranges reported in Turkish and global populations, within the normal ranges [2,28,29,30,31,32,33,34,35]. These descriptive characteristics indicate that the sample was not unusual in terms of reproductive characteristics. However, agreement on these characteristics alone does not guaranty that the sample was representative of the wider population of female university students. The high standard deviation for cycle length (±7.29 days) shows variation in cycle regularity, which is a common finding in young adults and related to anovulatory cycles and poor follicular development at the edges of reproductive lifespan [32,33].

Before the program, more than 50% of participants met the PMSS criteria for PMS. In addition, the percentage of those meeting the benchmark dropped from 58.9% to 49.1% following the intervention phase (not statistically significant). A meta-analysis evaluating the effects of psychosocial interventions on PMS severity noted that the combined effects of coping skills training and 11 other psychosocial interventions demonstrated statistically significant beneficial effects on PMS severity, whereas the other interventions exhibited no significant results [36]. In a recent study, Ozeki et al. administered an offline/online checklist-based PMS educational tool to 3090 females and monitored the effects 8 months later. The women’s premenstrual and menstrual symptom scores showed a trend toward improvement; however, this change did not reach statistical significance [37]. By contrast, a recent randomized controlled open-label trial in Omani adolescents with PMS revealed that a structured healthy-diet and motivational intervention significantly reduced PMS symptom severity and enhanced the quality of life [38]. The direction of this change is consistent with earlier research in the areas of educational and nutritional intervention (e.g., [39,40,41]) as discussed below, but the effect size is small and the confidence interval indicates the possibility of no change.

In the present study, PMS symptom severity scores significantly declined over the online education intervention period. Although this effect size is small, it is statistically significant (*p* = 0.001; d_z_ = 0.31; 95% CI 0.12–0.50). Likewise, a meta-analysis by Han et al. on the effects of psychosocial interventions observed that the overall effect size (SMD) was −0.29; by contrast, the effect was greater in more intensive coping skills training programs [36]. However, the estimate reported by Han et al. was based on controlled trials, whereas the estimate in the present study represents an uncontrolled pre–post change. Therefore, the two effect estimates are not directly comparable.

In the current study, statistically significant decreases were observed in the scores of the PMSS subdimensions of depressive mood, anxiety, fatigue, irritability, depressive thoughts, pain, sleep alterations, and bloating after the nutrition education program. All eight were significant below q = 0.05 after the Benjamini–Hochberg correction. In agreement with the more conservative Holm–Bonferroni method, results for fatigue, irritability and sleep changes did not reach the standard threshold (adjusted *p* = 0.122, 0.084 and 0.122, respectively) and thus these three outcomes should be considered provisional. This is important to point out that the depressive mood and bloating subdimensions showed the greatest standardized changes. However, the confidence intervals for the effect sizes of the subscales overlapped considerably. Thus, we cannot conclude that nutrition education has a greater effect on mood symptoms than on somatic symptoms related to diet, regardless of the fact that such a tendency is biologically plausible due to known relationships between diet composition, fluid retention, and premenstrual mood symptoms [25,42].

A systematic review of the effect of nutritional education on PMS concluded that evidence for these interventions is inconsistent and most of the studies have a high risk of bias [39]. A study of educational interventions conducted in adolescents over 4 weeks with four 30 min sessions reported a significant difference in the rates of moderate-to-severe PMS cases between the intervention and control groups during the follow-up period; however, these rates did not show a significant difference between the two groups at baseline [40]. A recent systematic review included 32 articles reporting 31 randomized controlled trials involving 3254 participants and revealed that nutritional interventions did not significantly change diet quality and food choice but consistently demonstrated significant positive effects on the psychological symptoms of PMS [39]. In another nutritional intervention study, although the educational intervention decreased total PMSS scores by 27.5 points, significant reductions were observed particularly in subscales, including depressive feelings, anxiety, irritability, depressive thoughts, appetite changes, and sleep disturbances [41].

In the present study, the appetite changes subscale of the PMSS exhibited no significant difference. This finding may be explained by the fact that appetite changes are influenced by knowledge levels, hormonal fluctuations during the luteal phase, reward systems, and mood changes. During the luteal phase, females experience an increase in energy intake and consumption of carbohydrate- and sugar-rich foods [43,44]. Moreover, hedonic hunger and emotional eating are positively associated with PMS severity [45,46]. From a hormonal standpoint, appetite changes may be associated with the estrogen–progesterone–serotonin–gamma-aminobutyric acid (GABA)–leptin/ghrelin–insulin axes and the reward response of the brain; therefore, a 6-week nutrition education program may have been too short to alter this domain [25,45,47,48].

Studies investigating the effects of nutrition education on knowledge level and eating attitudes in relation to PMS remain controversial. A study evaluating the effects of web-based education over 4 weeks on PMS reported that education delivered via WhatsApp increased the tendency to improve eating habits [49]. Similarly, studies have demonstrated that both face-to-face and online education increase knowledge levels among females with PMS [50,51,52]. Atoloye et al. reported a non-randomized study protocol for an intervention combining school-based cultivation of native vegetables and fruits with nutrition education via WhatsApp in southwest Nigeria; however, outcome data from this study are not yet available, and therefore its efficacy has not yet been established [53]. Similarly, a study on WhatsApp chatbot-based educational intervention targeting pregnant women underscored that the chatbot’s structured, interactive, and accessible approach to delivering information, which encourages active user participation through automated responses and instant feedback, strengthens knowledge retention and increases motivation to learn. This study also revealed that face-to-face education was equally effective as the chatbot [54]. Another randomized controlled trial demonstrated that nutrition education did not lead to improvements in healthy eating or quality of life among college students with PMS [38].

The present study did not find significant differences in the nutrition knowledge and eating attitude assessments of the participants following the 6-week training program where the confidence intervals for both change scores did not include effects greater than approximately 0.27 SD, indicating that great improvement in these areas is unlikely over this period and format. Furthermore, in the correlation analysis of changes in scale scores, the ΔPMSS and ΔNKLSA change scores exhibited no significant association. Another consideration is that the EAT-26 measures attitudes and behaviors related to disordered eating and not the adoption of a healthier diet; hence, a nutrition education intervention focused on daily eating habits would not be expected to change EAT-26 scores in a non-clinical population with a low baseline mean (10.68) and any lack of change in this measure should not be interpreted as a lack of change in dietary behavior. No assessment of dietary consumption or nutritional quality was performed, and this aspect was not investigated. The association between PMS and eating behaviors is bidirectional; although eating disorders can exacerbate PMS symptoms, PMS can also trigger emotional and uncontrolled eating [55]. Longer-term, follow-up, and structured studies are warranted to understand how dietary behaviors frequently observed in PMS may change [42].

Furthermore, studies have generally demonstrated significant effects in face-to-face or actively interactive web-based training programs. In the present study, information cards were shared via WhatsApp, and a less interactive study design was implemented. Although participants were provided a weekly interactive question-and-answer session, there was no demand from them. This suggests that a 6-week exposure to passively delivered material may be insufficient for knowledge acquisition. Studies investigating factors associated with educational psychology have indicated that testing/active recall is more effective than restudying in enhancing the ability to learn new information and that active recall practice is more effective than other learning methods [56,57]. These findings demonstrate that merely being passively exposed to information (e.g., reading, watching, and viewing flashcards) leads to significantly weaker and less lasting learning than methods that involve actively recalling information (question-and-answer sessions, quizzes, and tests).

This study examined whether the change in total PMSS scores differed according to tobacco use and BMI category, and nicotine use was associated with a higher burden of premenstrual symptoms. Obesity was also associated with premenstrual symptoms. A meta-analysis of 13 studies examining the relationship between PMS and smoking reported a higher risk and more severe premenstrual symptoms in women who smoked [58]. Similarly, in a case–control study by Fernández et al., it was found that smokers were more likely to develop PMS and PMDD. In this study, total PMSS scores decreased significantly in non-smoking participants, while the decrease was not statistically significant in tobacco users [59]. However, this finding should not be interpreted as evidence of a different intervention effect according to tobacco use. In this study, BMI was also associated with the burden of premenstrual symptoms, but the findings of studies in the literature are not entirely consistent [60,61]. In our subgroup analysis, a significant decrease in total PMSS scores was observed in participants with BMI < 18.5 kg/m^2^, while the decrease was not statistically significant in participants with BMI 18.5–24.9 kg/m^2^ and BMI ≥ 25.0 kg/m^2^. Given the small subgroup sizes and the exploratory nature of these analyses, these findings should be interpreted cautiously and confirmed by studies with sufficient power.

Finally, this study identified a weak but significant positive correlation between the ΔPMSS and ΔEAT-26 change scores, indicating that changes on the two scales tend to move in the same direction. This correlation is one of several exploratory analyses, explains about 3.5% of the variance, and would not survive adjustments for multiple comparisons, and thus should not be given significant interpretive importance and cannot be understood causally in either direction.

## 5. Strengths and Limitations

A major strength of this study is the use of validated tools in Turkish, reporting effect sizes with confidence intervals in addition to *p*-values, pre-specification of a single primary outcome and adjustment of the subscale family for multiple comparisons. However, this research had several major limitations. First, the single-arm pretest/posttest design without a control group did not allow to attribute the observed changes to the educational intervention and did not exclude the possibility of natural fluctuations of symptoms, regression toward the mean or a placebo/attention (Hawthorne) effect due to repeated self-reporting. Moreover, the PMSS is a retrospective single-timepoint assessment as opposed to the recommended prospective daily symptom recording across successive cycles for premenstrual disorder assessment [22], and because pre- and postintervention assessments were not performed in the same luteal-phase timing and cycle phase was not documented, the variability associated with cycle phase may have influenced the observed change and represents an important limitation when interpreting the primary outcome. Furthermore, exceeding the PMSS threshold does not imply that a clinical diagnosis is made; thus, the data in Table 3 show the percentage meeting a questionnaire criterion rather than the incidence of PMS. Fourth, the uniform (mostly individual undergraduate students) single-location convenience sample of participants limits generalizability of the results, and individuals with a higher symptom burden may have been more likely to participate. Sample size was sufficient for the primary PMSS outcome but not powered to detect small subscale effects and weak correlations. Subscale-level results with *p*-values near 0.05 (fatigue, irritability, and sleep alterations) do not survive Holm–Bonferroni correction and should be interpreted with caution. In addition, engagement on WhatsApp was not systematically recorded, and therefore the amount of education acquired cannot be correlated with results and non-compliance cannot be measured. Additionally, there was no evaluation of dietary intake or nutritional quality, and it is unclear if dietary habits changed in any way, as the EAT-26 measures disordered eating perspectives and cannot replace such an assessment. The graphics for the educational cards were created using a generative artificial intelligence tool, but all text was written by the research team and checked against source documents prior to dissemination, requiring specific check protocols for the use of such tools in intervention materials, as described in Section 2.6. Finally, the study period may not be enough to see changes in knowledge or dietary habits, and there were no follow-up assessments after the post-intervention assessment.

## 6. Conclusions

This study demonstrated that the 6-week WhatsApp-based online nutrition education program did not significantly improve the nutrition knowledge and general eating attitude of female college students. However, the self-reported severity of premenstrual symptoms was significantly reduced in the intervention period, especially in terms of mood-related and bloating symptoms. The magnitude of this change was small, the proportion of participants who met the PMSS cut-off was similar, and the study design was uncontrolled. Thus, the study provides preliminary evidence of a pre–post change in self-reported symptoms, not evidence that WhatsApp-based nutrition education is an effective intervention for managing PMS.

The findings suggest that nutrition education delivered through accessible digital platforms should be further evaluated as a complementary approach to PMS management. Future randomized controlled studies should include longer follow-up periods, standardized assessment according to cycle phase, assessment of dietary intake and adherence, and more interactive components.

## Figures and Tables

**Table 1:** Sociodemographic and menstrual characteristics of the participants.

Variables		*n*	%
Marital status	Single	106	94.6
	Married	6	5.4
Educational status	Two-year degree	20	17.9
	Bachelor’s degree	88	78.6
	Postgraduate degree	4	3.6
Tobacco use	Yes	31	27.7
	No	81	72.3
Alcohol use	Yes	18	16.1
	No	94	83.9
**Variables**		**Mean ± SD**	
Age (years)		22.76 ± 2.81	
BMI (kg/m^2^)		22.9 ± 3.74	
Daily water intake (L)		1.51 ± 0.66	
Number of main meals		2.18 ± 0.47	
Number of snacks		1.38 ± 0.85	
Age at menarche (years)		13.06 ± 1.26	
Menstrual cycle length (days)		28.97 ± 7.29	
Menstrual bleeding duration (days)		6.26 ± 1.22	

Abbreviations: BMI, body mass index; SD, standard deviation.

**Table 2:** Comparison of scale scores before and after the online nutrition education program.

Scale Scores	Pretest Mean ± SD	Posttest Mean ± SD	Mean Change	95% CI of Change	t	*p*	q (BH)	Cohen’s d_z_ (95% CI)
NKLSA total	53.20 ± 7.16	52.65 ± 6.18	0.54 ± 6.95	−0.76 to 1.84	0.829	0.409	—	0.08 (−0.11 to 0.27)
EAT-26 total	10.68 ± 9.60	11.16 ± 10.48	−0.48 ± 11.35	−2.61 to 1.65	−0.450	0.654	—	−0.04 (−0.23 to 0.15)
PMSS total	146.43 ± 35.46	132.15 ± 40.13	14.28 ± 46.34	5.60 to 22.96	3.261	0.001	—	0.31 (0.12 to 0.50)
Depressive mood	24.92 ± 5.67	22.26 ± 6.67	2.66 ± 7.04	1.34 to 3.98	3.998	<0.001	0.001	0.38 (0.19 to 0.57)
Anxiety	19.80 ± 7.14	17.66 ± 6.88	2.14 ± 8.59	0.53 to 3.75	2.640	0.009	0.017	0.25 (0.06 to 0.44)
Fatigue	21.36 ± 5.55	19.94 ± 6.63	1.42 ± 7.30	0.05 to 2.79	2.057	0.042	0.047	0.19 (0.01 to 0.38)
Irritability	17.68 ± 5.17	16.03 ± 5.88	1.65 ± 7.45	0.26 to 3.04	2.346	0.021	0.031	0.22 (0.03 to 0.41)
Depressive thoughts	21.30 ± 7.48	18.82 ± 7.94	2.48 ± 9.18	0.76 to 4.20	2.863	0.005	0.011	0.27 (0.08 to 0.46)
Pain	10.33 ± 3.15	9.04 ± 3.55	1.29 ± 4.28	0.49 to 2.09	3.200	0.002	0.006	0.30 (0.11 to 0.49)
Appetite changes	10.60 ± 3.21	10.29 ± 3.61	0.30 ± 3.99	−0.45 to 1.05	0.805	0.423	0.428	0.08 (−0.11 to 0.26)
Sleep changes	9.60 ± 3.51	8.76 ± 3.44	0.84 ± 4.29	0.04 to 1.64	2.069	0.041	0.047	0.20 (0.01 to 0.38)
Bloating	10.84 ± 3.31	9.36 ± 3.71	1.48 ± 4.43	0.65 to 2.31	3.544	0.001	0.003	0.33 (0.15 to 0.52)

t: paired samples *t*-test; CI: confidence interval; q (BH): Benjamini–Hochberg false discovery rate–adjusted *p*-value, computed within the family of the nine PMSS subscales only. Cohen’s d_z_ = mean of the within-person difference scores/SD of the difference scores. The PMSS total score is the primary outcome and is reported unadjusted.

**Table 3:** Proportion of participants meeting the PMSS threshold for PMS before and after the online nutrition education program.

PMSS Threshold Status	Pretest		Posttest		*p*
	*n*	%	*n*	%	
Below threshold	46	41.1	57	50.9	0.108
At or above threshold (≥132)	66	58.9	55	49.1	

*p*: McNemar exact test. Exceeding the PMSS cut-off at a single assessment is not equivalent to a clinical diagnosis of a premenstrual disorder.

**Table 4:** Association between changes in scale scores before and after the online nutrition education program (exploratory).

Variable	ΔPMSS	ΔNKLSA
ΔPMSS	1	0.001 (0.994)
ΔNKLSA	0.001 (0.994)	1
ΔEAT-26	0.187 (0.048)	0.057 (0.547)

Abbreviations: ΔPMSS: change in Premenstrual Syndrome Scale score, ΔNKLSA: change in Nutrition Knowledge Level Scale for Adults score, ΔEAT-26: change in Eating Attitudes Test-26 score. Values are Pearson r (*p*). These analyses are exploratory and were not corrected for multiplicity.

**Table 5:** Exploratory subgroup analysis of the change in PMSS total score by tobacco use and BMI category.

Subgroup	*n*	PMSS Pretest	PMSS Posttest	Mean Change (95% CI)	*p*	d_z_
Tobacco use: yes	31	151.84 ± 35.74	132.52 ± 43.27	19.32 (−1.30 to 39.95)	0.065	0.34
Tobacco use: no	81	144.36 ± 35.35	132.01 ± 39.14	12.35 (3.02 to 21.67)	0.010	0.29
BMI < 18.5 kg/m^2^	13	161.54 ± 35.16	125.31 ± 40.97	36.23 (7.22 to 65.24)	0.019	0.75
BMI 18.5–24.9 kg/m^2^	77	141.61 ± 36.68	132.12 ± 40.12	9.49 (−0.39 to 19.38)	0.059	0.22
BMI ≥ 25.0 kg/m^2^	22	154.36 ± 27.91	136.32 ± 40.99	18.05 (−5.31 to 41.40)	0.123	0.34

Mean change was calculated as pre-test minus post-test; therefore, positive values indicate a reduction in PMSS total score. d_z_: Cohen’s d_z_ for paired samples; CI: confidence interval; BMI: body mass index; PMSS: Premenstrual Syndrome Scale.

## Data Availability

The data obtained in this study are available from the corresponding author upon request.

## Abbreviations

PMS	Premenstrual syndrome
EAT-26	Eating Attitudes Test-26
NKLSA	Nutrition Knowledge Level Scale for Adults
PMSS	Premenstrual Syndrome Scale

## Appendix A

**Table A1:** Weekly themes, learning objectives and card counts for the 6-week nutrition education programme.

Week	Theme	Learning Objectives	Cards
Week 1	Principles of adequate and balanced nutrition	Describe the components of an adequate and balanced diet; identify the main food groups and recommended portions.	7
Week 2	The menstrual cycle and nutritional requirements across its phases	Describe the follicular, ovulatory and luteal phases; explain how energy and nutrient needs vary across the cycle.	7
Week 3	Micronutrients reported in relation to premenstrual symptoms	Identify calcium, magnesium, vitamin B6 and vitamin D food sources and the evidence linking them to premenstrual symptoms.	7
Week 4	Dietary patterns, meal regularity and meal skipping	Recognise the relationship between meal regularity, blood glucose stability and premenstrual symptom severity.	7
Week 5	Nutrition in the premenstrual and menstrual phases; management of specific symptoms	List practical dietary strategies for bloating, fatigue, mood symptoms, pain and sleep disturbance.	7
Week 6	Consolidation, practical menu planning and frequently asked questions	Construct a one-day sample menu consistent with the principles covered; correct common misconceptions.	7
Total			42

One flashcard was shared per day for 42 consecutive days. Content was written by three author-dietitians on the basis of references [22,23,24,25] and independently checked for scientific accuracy before delivery.
